# Strengthening gestational diabetes mellitus prevention, diagnosis, and care in Africa: evidence and policy priorities from a systematic review, meta-analysis, and bibliometric analysis

**DOI:** 10.3389/fpubh.2026.1953914

**Published:** 2026-09-18

**Authors:** Benjamin Aye Simon, Lawrence Chauke, Nalini Govender, Chester Kalinda, Francis Akpa-Inyang, Yasmeen Thandar

**Affiliations:** 1 Department of Basic Medical Sciences, Faculty of Health Sciences, Durban University of Technology, Durban, South Africa; 2 Durban University of Technology, Durban, South Africa; 3 University of the Witwatersrand Johannesburg, Johannesburg, South Africa; 4 Department of Obstetrics and Gynaecology, Faculty of Health Sciences, The University of the Witwatersrand, Johannesburg, South Africa; 5 School of Global Health, University of Global Health Equity (UGHE), Kigali, Rwanda; 6 Department of Public Health, College of Health Sciences, School of Medicine, University of KwaZulu-Natal, Durban, South Africa

**Keywords:** Africa, diabetes prevention and management, gestational diabetes mellitus, health policy and practice, health systems strengthening, maternal health, non-communicable diseases, pregnancy diabetes

## Abstract

Gestational diabetes mellitus (GDM) is an increasing public health challenge in Africa, contributing substantially to maternal and neonatal morbidity. Evidence from our systematic review, meta-analysis, and bibliometric analysis shows that inconsistent screening practices, reliance on selective rather than universal testing, and limited health system capacity contribute to underdiagnosis and poor pregnancy outcomes. GDM is associated with adverse maternal and neonatal complications and increases the long-term risk of type-2 diabetes for mothers and offspring. Our bibliometric findings reveal uneven research capacity, limited regional collaboration, and evidence gaps across Africa. We propose five policy priorities: standardising GDM screening, strengthening prevention, expanding access to diagnostics and treatment, improving workforce capacity and follow-up, and increasing investment in African-led research to improve maternal health and reduce the intergenerational burden of diabetes.

## 1 Background and problem statement

Gestational diabetes mellitus (GDM) is an important contributor to maternal and neonatal morbidity in Africa. It is also an increasingly important public health concern in Africa, reflecting broader epidemiologic transitions marked by rising obesity, urbanisation, and changing lifestyles. Current evidence indicates that approximately 14% of pregnancies in sub-Saharan Africa are affected, with country-level estimates ranging widely depending on population characteristics and diagnostic criteria used (1–3). Rising obesity, advanced maternal age, family history of diabetes, and prior GDM are consistently identified as risk factors, while health system constraints limit early detection and effective management, thereby contributing to poor outcomes (1, 4, 5).

Findings from our systematic review and meta-analysis indicate marked variation in screening and diagnostic practices, with many countries continuing to use selective and risk-based screening rather than universal oral glucose tolerance test (OGTT), leading to underdiagnosis and missed opportunities to prevent adverse outcomes (1). GDM is strongly associated with macrosomia, preeclampsia, stillbirth, and higher rates of caesarean delivery, a higher risk of type-2 diabetes and obesity in both mothers and their children (2, 5). Evidence further shows that improved glycaemic control through lifestyle modification and pharmacologic treatment can significantly reduce these risks (3).

Evidence synthesized from our systematic review, meta-analysis, and bibliometric analysis of 44 African GDM prevalence studies published between 1999 and 2024 indicates a high and growing burden of GDM, with a pooled prevalence of 12.62% across the full period, increasing to 13.43% between 2014–2024 and 14.52% between 2020–2024 (Freeman-Tukey random-effects models) (1). These estimates are consistent with regional reports indicating that GDM affects nearly one in seven pregnancies in some settings, highlighting its growing public health importance (2, 3, 6).

Across diverse African populations, GDM is consistently linked to both modifiable and non-modifiable risk factors. Frequently reported determinants include advanced maternal age, overweight and obesity, family history of diabetes, multiparity, and previous adverse obstetric outcomes, particularly a history of GDM or delivery of a macrosomic infant (4, 5, 7). Urbanisation, declining physical activity, and dietary shifts toward energy dense foods further increase the risk (1). In Southern Africa, additional region-specific influences such as HIV infection and antiretroviral therapy have also been documented (8, 9).

The maternal and neonatal consequences of GDM are considerable. Affected women experience higher rates of preeclampsia, gestational hypertension, caesarean delivery, preterm birth, and labour complications (1). Infants face increased risks of macrosomia, neonatal hypoglycaemia, respiratory distress, admission to neonatal intensive care, and stillbirth, particularly in low resource settings (5, 6, 10).

Despite this public health burden, prevention, diagnosis, and management of GDM remain inadequate. Screening and diagnostic practices differ widely, with reliance on risk-based approaches and inconsistent use of OGTT resulting in underdiagnosis, especially among younger or non-obese women (1–3, 11). Constraints in laboratory capacity, financial barriers, and weak postpartum follow-up further limit effective care, underscoring the urgent need for strengthened and context appropriate policies to reduce preventable maternal and neonatal morbidity associated with GDM in Africa. Building on the evidence generated from our published systematic review, meta-analysis, and bibliometric analysis paper (1), the current paper synthesises epidemiological findings, health system challenges, and patterns of research production to identify evidence-informed strategies and actionable policy priorities for strengthening the prevention, diagnosis, and care of GDM in Africa. The overarching goal is to support policymakers, programme implementers, clinicians, and researchers in developing coordinated, context-specific approaches that improve maternal and neonatal health outcomes while reducing the long-term burden of diabetes across the continent.

## 2 Policy options and implications

### 2.1 Evidence from the systematic review and meta-analysis

The findings from our systematic review and meta-analysis provide a robust evidence base for strengthening GDM prevention, diagnosis, and care across Africa (1). Collectively, the evidence demonstrates that rising disease burden, marked variation in diagnostic practices, persistent health-system constraints, and uneven research capacity require coordinated policy responses that extend beyond clinical management to encompass prevention, health-system strengthening, surveillance, and research investment (1). Building on these findings, Figure 1 presents an Integrated Policy Framework for Strengthening GDM Prevention, Diagnosis, and Care in Africa, while the policy options below translate the evidence into actionable priorities for policymakers, health systems, programme implementers, clinicians, researchers, and development partners (1).

**Figure 1:**
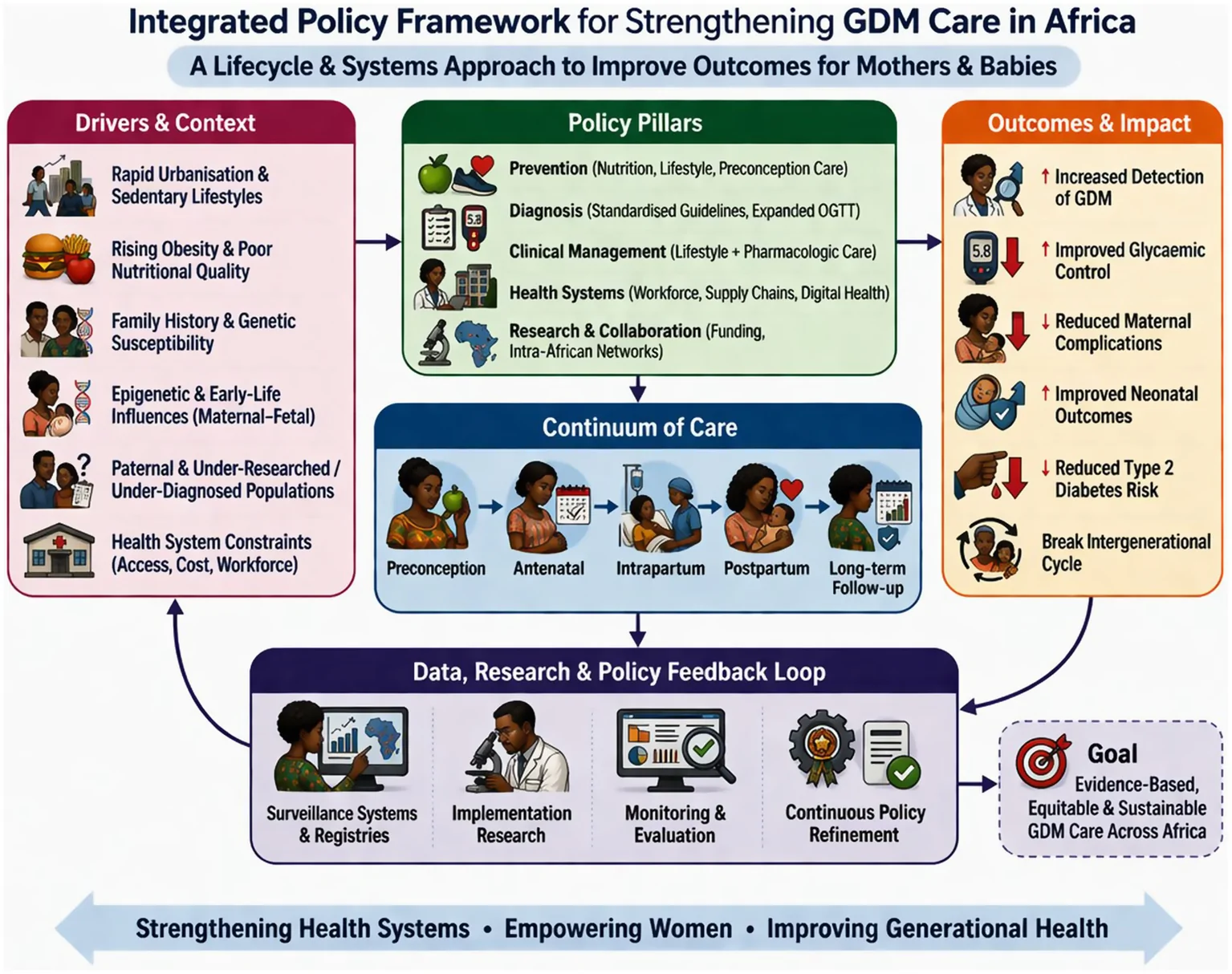
Life-course integrated policy framework for strengthening GDM prevention, diagnosis, and care in Africa.

#### 2.1.1 Policy option 1: harmonise GDM screening and diagnostic guidelines across Africa

The systematic review and meta-analysis identified substantial heterogeneity in GDM prevalence estimates, largely driven by differences in diagnostic criteria and screening strategies, including the use of WHO (1999), WHO (2013), IADPSG, ADA, and NICE recommendations (1, 3, 7, 12). This inconsistency compromises data comparability, limits surveillance, and contributes to variations in clinical practice across and within countries. Although universal screening using the 75 g OGTT consistently detects more women with GDM than selective or risk-based screening, implementation remains constrained by limited laboratory infrastructure, workforce shortages, and financial barriers (1, 2, 11).

National governments and regional health bodies should prioritise the adoption of harmonised, context-appropriate GDM screening and diagnostic guidelines, with the IADPSG criteria, as endorsed by the WHO (2013) recommendations, serving as the preferred reference standard where feasible. The IADPSG criteria have demonstrated greater sensitivity for identifying GDM than older diagnostic thresholds and facilitate earlier detection of women at risk of adverse maternal and neonatal outcomes, while also improving comparability of epidemiological data across settings (1, 3, 7, 12). Universal screening using a 75 g OGTT at 24–28 weeks’ gestation should be progressively implemented as the standard of care, with phased implementation strategies adopted in resource-constrained settings to strengthen laboratory capacity, workforce training, and quality assurance. Standardised diagnostic protocols would improve case detection, strengthen surveillance systems, facilitate regional comparisons, and promote equitable access to high-quality maternal healthcare across Africa (1–3, 7, 11, 12).

#### 2.1.2 Policy option 2: prioritise prevention through integrated maternal and non-communicable disease (NCD) programmes

The evidence consistently identifies overweight and obesity, advanced maternal age, unhealthy dietary patterns, physical inactivity, delayed antenatal booking, and socioeconomic disadvantage as major determinants of GDM across African populations (1, 3–5, 13, 14). These findings demonstrate that many drivers of GDM are preventable and closely linked to the continent’s ongoing nutritional and epidemiological transition.

##### 2.1.2.1 Policy implications

Prevention should become a core component of national maternal health and NCD policies. Integrating preconception counselling, healthy weight promotion, nutrition education, physical activity interventions, and early antenatal engagement into existing reproductive and maternal health programmes would reduce GDM risk while simultaneously addressing broader metabolic diseases. Such an approach offers substantial long-term health and economic benefits by reducing complications during pregnancy and preventing future type-2 diabetes among mothers and their children (1, 6, 15).

#### 2.1.3 Policy option 3: strengthen primary healthcare capacity for equitable GDM care

Our review demonstrates that access to GDM diagnosis and management remains concentrated in tertiary urban facilities, whereas primary and rural healthcare settings frequently lack laboratory capacity, glucose monitoring supplies, trained personnel, nutrition services, and effective referral systems (1, 4, 5, 16). These disparities contribute to delayed diagnosis, fragmented care, and poorer maternal and neonatal outcomes.

##### 2.1.3.1 Policy implications

Policymakers should strengthen primary healthcare systems as the foundation for equitable GDM care. Investments should prioritise workforce training, affordable diagnostic technologies, essential medicines, nutritional counselling services, digital clinical decision-support tools, and functional referral pathways. Expanding GDM services beyond tertiary hospitals would improve early detection and management while reducing geographical and socioeconomic inequities in maternal healthcare (1, 4, 5).

#### 2.1.4 Policy option 4: establish life-course models of care beyond pregnancy

The systematic review shows that GDM is associated with significant maternal and neonatal complications and substantially increases women’s future risk of type-2 diabetes, while exposing offspring to elevated lifetime metabolic risk (1, 2, 6, 10, 11, 15). Despite this, structured postpartum glucose testing and long-term follow-up remain poorly implemented across Africa (1, 6, 7, 15).

##### 2.1.4.1 Policy implications

National policies should reposition GDM as a life-course condition rather than a pregnancy-limited complication. Integrating postpartum diabetes screening, long-term metabolic surveillance, lifestyle support, and family-centred counselling into routine maternal and primary healthcare would strengthen continuity of care and help interrupt the intergenerational transmission of diabetes and other metabolic disorders (1, 6, 7, 15). Although the life-course approach to GDM is recognised globally, our proposed framework contextualises this perspective within the realities of African healthcare systems by directly addressing the health-system constraints, gaps in long-term follow-up, and intergenerational consequences of GDM identified in our systematic review, meta-analysis, and bibliometric analysis. As illustrated in Figure 1, this continuum of care should extend from preconception through pregnancy, childbirth, postpartum follow-up, and long-term prevention.

#### 2.1.5 Policy option 5: strengthen health information systems and routine surveillance

The systematic review and bibliometric analysis identified substantial weaknesses in routine GDM data collection, inconsistent reporting practices, and poor integration of antenatal, delivery, and postpartum records, limiting accurate estimation of disease burden and policy evaluation (1, 6).

##### 2.1.5.1 Policy implications

Governments should integrate standardised GDM indicators into national health information systems and strengthen digital surveillance platforms capable of linking maternal care across the continuum of pregnancy and beyond (Figure 1). Improved routine data would enable more accurate burden estimation, facilitate programme monitoring, support evidence-based resource allocation, and strengthen accountability for maternal health outcomes (1).

### 2.2 Evidence from the bibliometric analysis

The bibliometric analysis demonstrates that African GDM research expanded substantially between 1999 and 2024, particularly after 2013, but remains geographically concentrated, dominated by descriptive epidemiology, and characterised by limited intra-African collaboration (1). Important gaps persist in intervention research, postpartum outcomes, implementation science, health-system research, and economic evaluation (1). Similar deficiencies have been identified across African diabetes research, where intervention studies remain concentrated in relatively few countries and urban settings, with limited evidence from primary healthcare and poorer-resource settings (17). These findings indicate that increasing publication volume alone is insufficient; investment must strengthen research capacity, regional collaboration, harmonised data systems, and translation of evidence into policy.

#### 2.2.1 Policy option 6: invest in research capacity and Pan-African collaboration

Research productivity remains concentrated in a small number of countries and institutions, with South Africa providing substantial scientific leadership, while several African regions remain under-represented (1). International partnerships are stronger than intra-African collaboration, limiting multicountry research and development of regionally representative evidence (1).

##### 2.2.1.1 Policy implications

Governments, research councils, universities, regional organisations, and development partners should prioritise sustainable research infrastructure, researcher training, multicountry studies, and African-led collaborative networks. Funding mechanisms should incentivise equitable partnerships between established and emerging institutions and strengthen epidemiological, biostatistical, implementation-science, and health-economic capacity. Regional GDM research hubs linked to existing centres of excellence could facilitate shared expertise, harmonised research methods, mentorship, and multicountry studies while strengthening African scientific leadership. Strengthening such networks would broaden the geographical representation of GDM evidence and create the research capacity required to respond to emerging maternal metabolic health challenges across Africa (1).

#### 2.2.2 Policy option 7: establish harmonised Pan-African GDM research and data platforms

Differences in diagnostic criteria, study designs, outcome definitions, and reporting practices limit comparability and evidence synthesis across African countries (1). Earlier African systematic reviews similarly identified substantial variation in GDM screening and diagnostic approaches and major geographical evidence gaps (18, 19).

##### 2.2.2.1 Policy implications

Regional bodies and national health authorities should establish a harmonised minimum dataset for GDM research and surveillance, incorporating diagnostic methods, maternal risk factors, treatment, pregnancy and neonatal outcomes, and postpartum follow-up. Standardised definitions and interoperable data systems would enable multicountry analyses, improve disease surveillance, facilitate external validation of risk-prediction models, and strengthen evidence-based resource allocation.

#### 2.2.3 Policy option 8: redirect research investment towards implementation, intervention and health-economic evidence

African GDM research remains strongly focused on prevalence, risk factors, and diagnosis, while intervention, implementation, long-term follow-up, and economic evidence remain comparatively limited (1). This gap is particularly important because African diabetes intervention research remains poorly representative of many countries and rural and primary-care settings (17). Economic evaluations also show that the value of GDM screening and management varies according to diagnostic strategies, population characteristics, intervention effectiveness, and healthcare costs, underscoring the importance of context-specific analyses (1, 20–22).

##### 2.2.3.1 Policy implications

Funding should increasingly support pragmatic intervention studies, implementation research, postpartum continuity-of-care strategies, and integration of GDM services into primary healthcare and NCD programmes. Cost-effectiveness, budget-impact, affordability, and resource-allocation analyses should accompany these studies to identify interventions that provide the greatest health benefit within constrained healthcare budgets.

#### 2.2.4 Policy option 9: create a research–surveillance–policy feedback mechanism

Growing research output will have limited population impact unless evidence is systematically translated into clinical guidelines, programmes, and policy (1).

##### 2.2.4.1 Policy implications

Governments and regional organisations should establish formal knowledge-translation platforms linking researchers, clinicians, policymakers, programme implementers, and communities. The research–surveillance–policy feedback loop presented in Figure 1 would allow emerging evidence to inform policy, surveillance to evaluate implementation, and identified gaps to guide subsequent research. This continuous learning approach could transform Africa’s expanding GDM research base into responsive, evidence-informed improvements in prevention, diagnosis, and care.

## 3 Actionable policy recommendations

### 3.1 Strengthen prevention

Preventing GDM in Africa requires a shift toward earlier and population-level interventions that address modifiable risk factors before and during pregnancy. Community-based education programs should be implemented and scaled up to improve awareness of GDM, its risk factors, and its consequences for maternal and neonatal health. Evidence from the review shows that overweight, obesity, poor diet, and physical inactivity are among the strongest and most consistent predictors of GDM across African settings (3, 5). Nutrition and lifestyle counselling should be integrated into existing maternal, adolescent, and reproductive health platforms to promote healthy behaviours before and during pregnancy. Where feasible, preconception screening for high-risk women should be incorporated into routine reproductive health services to enable early risk stratification and targeted counselling (4, 7). These could include BMI thresholds, detailed family history of diabetes, previous GDM diagnosis, or specific biomarkers known to predict GDM risk.

### 3.2 Improve diagnosis

Improving diagnosis is central to reducing missed GDM cases. Countries should move toward standardised national screening protocols aligned with WHO/IADPSG thresholds, while allowing context-specific adaptations where laboratory capacity is limited. Evidence from multiple African studies shows that universal OGTT screening identifies more GDM cases than selective or risk-based approaches, which miss a substantial proportion of affected women (2, 3, 11). Governments should expand access to the 75 g OGTT through decentralisation of laboratory services, task-shifting, and strengthened referral systems is therefore essential. Screening should be fully integrated into routine antenatal care (ANC) visits, with clear guidance on timing and follow-up to ensure consistent implementation across all levels of care.

### 3.3 Enhance clinical management

Clinical management of GDM must be strengthened through timely diagnosis and access to appropriate treatment. Medical nutrition therapy (MNT) should be integrated into routine GDM care, with individualized dietary counselling delivered to support optimal glycaemic control. Universal access to timely GDM diagnosis through routine ANC should be prioritised to improve early detection and reduce underdiagnosis. Health systems should ensure the consistent availability of insulin and metformin, which are the most commonly used pharmacologic therapies in African studies, to optimise glycaemic control and reduce maternal and neonatal complications (3, 11). Clinical management protocols should also promote regular glucose monitoring, patient education, and appropriate follow-up to improve treatment adherence and pregnancy outcomes.

### 3.4 Strengthen health systems

Health system strengthening is required to sustain GDM services. Governments and health systems should build the capacity of frontline healthcare workers through training in GDM screening, diagnosis, glycaemic monitoring, counselling, and appropriate referral to improve the quality and consistency of care. Reliable supply chains for diagnostics, medications, and glucose monitoring equipment should be established and maintained. Clear referral pathways between primary, secondary, and tertiary facilities should be strengthened to address disparities between urban and rural settings documented in the literature review (1, 4, 5). Digital health solutions, including electronic ANC records and reminder systems, should be adopted to improve screening uptake, continuity of care, and follow-up. Structured postpartum follow-up programmes should be established to ensure continuity of care and facilitate long-term monitoring of women at increased risk of developing type-2 diabetes. National health information systems should incorporate standardised GDM indicators to improve surveillance, enable monitoring of trends, and support evidence-based planning (6).

### 3.5 Boost research and funding

Finally, sustained progress depends on greater investment in maternal health research. Our bibliometric analysis highlights the strong concentration of research output in a few countries and institutions, underscoring the need to support under-represented regions and promote intra-African collaboration (1). Governments, funding agencies, and development partners should prioritise investments in longitudinal studies, implementation research, and national or regional GDM registries to address gaps in postpartum follow-up, long-term outcomes, and the cost-effectiveness of screening and treatment strategies (1, 6, 7). Funding mechanisms should also strengthen African-led collaborative research networks and implementation science to accelerate the translation of evidence into policy and practice across the continent.

In summary, the evidence presented highlights the need for coordinated investment and stronger policy engagement to translate research into practice. Taken together, the findings underscore five priority actions to strengthen the prevention, diagnosis, and management of GDM across Africa:

- Strengthening prevention through nutrition, lifestyle, and preconception interventions
- Standardising GDM screening and diagnostic guidelines within routine ANC
- Expanding access to OGTT and essential medications, including MNT, insulin, and metformin
- Building health worker capacity, access to adequate equipment, and improving referral and follow up systems
- Increasing funding for maternal health research and intra-African collaboration

Taken together, these evidence-informed priorities provide a clear roadmap for reducing preventable maternal and neonatal complications, strengthening health systems, and disrupting the intergenerational vicious cycle of diabetes and obesity in Africa.

## 4 Implementation considerations

Effective implementation of strengthened GDM policies in Africa must be grounded in feasibility, affordability, and phased timelines, given existing health system constraints identified in the systematic review, meta-analysis, and bibliometric analysis.

### 4.1 Feasibility

- Integrating GDM screening into existing ANC platforms is feasible, as most African studies reported routine ANC attendance but inconsistent metabolic screening, indicating opportunities for service integration without creating parallel systems (4, 5).
- Task-shifting to trained nurses and midwives for risk assessment, glucose testing, and counselling can expand coverage in primary and rural facilities where specialist care is limited (2).

### 4.2 Cost implications

- Universal OGTT screening has higher upfront costs, but evidence shows it detects substantially more GDM cases than selective screening, potentially reducing downstream costs associated with preeclampsia, caesarean delivery, neonatal intensive care admissions, and stillbirth (2, 11).
- Use of metformin, where clinically appropriate, offers a lower-cost pharmacologic option compared with insulin and has already been adopted in several African settings (3, 5).
- Leveraging existing laboratory infrastructure and digital ANC registers can minimise additional system costs (6).

### 4.3 Timelines

- Short term (1–2 years): Harmonise national screening guidelines, train ANC providers, and include GDM indicators in routine health information systems.
- Medium term (3–5 years): Expand OGTT access beyond tertiary facilities, strengthen referral pathways, and scale community-based prevention programs targeting obesity and physical inactivity.
- Long term (5 + years): Establish national or regional GDM registries, support longitudinal follow-up of affected women, and embed GDM prevention within broader NCDs strategies.

These phased actions align with the evidence base and provide a realistic pathway for translating research into sustained improvements in maternal and neonatal outcomes across Africa.

## 5 Conclusion

Gestational diabetes mellitus represents an urgent and growing public health challenge in Africa, driven by rising obesity, changing lifestyles, and demographic transitions across the continent. Evidence synthesized from our systematic review and meta-analysis demonstrates wide but consistently increasing GDM prevalence, alongside substantial variation in screening and diagnostic practices that masks the true burden of disease. Our findings further confirm strong associations between GDM and adverse maternal and neonatal outcomes, including macrosomia, preeclampsia, stillbirth, and elevated long-term risks of type-2 diabetes for both mothers and their offspring. Complementing these findings, our bibliometric analysis highlights uneven research productivity concentrated in a few African countries and institutions, limited intra-African collaboration, and critical gaps in longitudinal and implementation research, underscoring the need for greater investment in context-specific evidence generation and policy translation. Addressing these challenges requires coordinated policy action to standardize GDM screening and diagnostic guidelines, strengthen prevention through nutrition, lifestyle, and preconception interventions, expand access to OGTT and essential treatments, build health workforce capacity, improve referral and postpartum follow-up systems, and increase funding for African-led maternal health research and regional collaboration. Implementing these evidence-informed strategies offers a critical opportunity to reduce preventable maternal and neonatal complications, strengthen health systems, and interrupt the intergenerational cycle of diabetes and obesity across Africa.

The strength of this study lies in its integration of systematic review, meta-analysis, and bibliometric analysis, providing a comprehensive assessment of both the epidemiological evidence and evolution of GDM research across Africa. However, the strength and generalisability of the evidence are tempered by substantial between-study and between-country heterogeneity in diagnostic criteria, screening strategies, population characteristics, and healthcare infrastructure. Furthermore, the inclusion of only English-language publications may have excluded relevant evidence from Francophone, Lusophone, and other non-English-speaking African settings, potentially contributing to the uneven geographical representation and limited evidence from several regions. These considerations warrant cautious interpretation of the pooled findings, which should be viewed as descriptive summaries of the available evidence rather than precise estimates applicable uniformly across African populations and settings.
